# Elevated level of lysophosphatidic acid among patients with HNF1B mutations and its role in RCAD syndrome: a multiomic study

**DOI:** 10.1007/s11306-022-01873-z

**Published:** 2022-02-18

**Authors:** Beata Małachowska, Justyna Janikiewicz, Karolina Pietrowska, Krystyna Wyka, Joanna Madzio, Kamila Wypyszczak, Marcin Tkaczyk, Sławomir Chrul, Rafał Zwiech, Anna Hogendorf, Maciej T. Małecki, Maciej Borowiec, Adam Krętowski, Wojciech Młynarski, Agnieszka Dobrzyń, Michał Ciborowski, Wojciech Fendler

**Affiliations:** 1 Department of Biostatistics and Translational Medicine, Medical University of Lodz, 15 Mazowiecka Street, 92-215 Lodz, Poland; 2 Laboratory of Cell Signaling and Metabolic Disorders, Nencki Institute of Experimental Biology PAS, 3 Pasteur Street, 02-093 Warsaw, Poland; 3 Clinical Research Centre, Medical University of Bialystok, 24a Sklodowska-Curie Street, 15-276 Bialystok, Poland; 4 Department of Pediatrics, Oncology and Hematology, Medical University of Lodz, 36/50 Sporna Street, 91-738 Lodz, Poland; 5 Department of Pediatrics, Immunology and Nephrology, Polish Mother’s Memorial Hospital Research Institute, 281/289 Rzgowska Street, 93-338 Lodz, Poland; 6 Department of Kidney Transplantation/Dialysis Department, Barlicki Memorial Teaching Hospital No. 1, Medical University of Lodz, 22 Kopcinskiego Street, 90-153 Lodz, Poland; 7 Department of Pediatrics, Diabetology, Endocrinology, and Nephrology, Medical University of Lodz, 36/50 Sporna Street, 91-738 Lodz, Poland; 8 Department of Metabolic Diseases, Medical College, Jagiellonian University, 2 Jakubowskiego Street, 30-688 Cracov, Poland; 9 Department of Clinical Genetics, Medical University of Lodz, 251 Pomorska Street, 92-213 Lodz, Poland; 10 Department of Endocrinology, Diabetology, and Internal Medicine, Medical University of Bialystok, 24a Sklodowska-Curie Street, 15-276 Bialystok, Poland; 11 Department of Radiation Oncology, Dana-Farber Cancer Institute, 450 Brookline Avenue, Boston, MA 02215 USA; 12 Department of Pediatrics Nephrology and Immunology, Medical University of Lodz, 281/289 Rzgowska Street, 93-338 Lodz, Poland

**Keywords:** Biomarkers, HNF1B, Maturity-onset diabetes of the young, Metabolomics, MODY, Pathogenesis, Polycystic kidney disease

## Abstract

**Introduction:**

Patients with hepatocyte nuclear factor-1 beta (*HNF1B*) mutations present a variable phenotype with two main symptoms: maturity onset diabetes of the young (MODY) and polycystic kidney disease (PKD).

**Objectives:**

Identification of serum metabolites specific for *HNF1B*mut and evaluation of their role in disease pathogenesis.

**Methods:**

We recruited patients with *HNF1B*mut (N = 10), *HNF1A*mut (N = 10), PKD: non-dialyzed and dialyzed (N = 8 and N = 13); and healthy controls (N = 12). Serum fingerprinting was performed by LC-QTOF-MS. Selected metabolite was validated by ELISA (enzyme-linked immunosorbent assay) measurements and then biologically connected with *HNF1B* by in silico analysis. HepG2 were stimulated with lysophosphatidic acid (LPA) and *HNF1B* gene was knocked down (kd) by small interfering RNA. Transcriptomic analysis with microarrays and western blot measurements were performed.

**Results:**

Serum levels of six metabolites including: arachidonic acid, hydroxyeicosatetraenoic acid, linoleamide and three LPA (18:1, 18:2 and 20:4), had AUC (the area under the curve) > 0.9 (*HNF1B*mut vs comparative groups). The increased level of LPA was confirmed by ELISA measurements. In HepG2^HNF1Bkd^ cells LPA stimulation lead to downregulation of many pathways associated with cell cycle, lipid metabolism, and upregulation of steroid hormone metabolism and Wnt signaling. Also, increased intracellular protein level of autotaxin was detected in the cells. GSK-3alpha/beta protein level and its phosphorylated ratio were differentially affected by LPA stimulation in *HNF1B*kd and control cells.

**Conclusions:**

LPA is elevated in sera of patients with *HNF1B*mut. LPA contributes to the pathogenesis of *HNF1B*-MODY by affecting Wnt/GSK-3 signaling.

**Supplementary Information:**

The online version contains supplementary material available at 10.1007/s11306-022-01873-z.

## Introduction

Maturity onset diabetes of the young (MODY) is a monogenic form of hereditary diabetes with a broad phenotype spectrum depending on its molecular culprit. It is a rare disease estimated to affect up to 1 in 13,000 children (Małachowska et al., 2018) and 1 to 10,000 adults (Owen, 2014). Hepatocyte nuclear factor-1 beta (*HNF1B*)-MODY, also called RCAD (renal cysts and diabetes syndrome) or MODY 5, is characterized by a variable coexistence of diabetes and congenital abnormalities of kidneys and urinary tract (CAKUT). It is caused by an autosomal dominant mutation in *HNF1B* gene. *HNF1B* is the Pit-1/Oct-1/Unc-86 (POU)-homeodomain transcription factor that regulates tissue-specific gene expression and embryonic development of liver, kidney, intestine, pancreas, and genitourinary system (Igarashi et al., 2005). It can bind to promoter DNA of target genes as a homodimer or heterodimer (with *Hnf1a*) leading to gene expression activation (mostly) or repression (rarely) (Musetti et al., 2014).

*HNF1B-*MODY is very rare and constitutes 1–5% cases of all MODY patients (Owen, 2014). There is phenotypic variability of *HNF1B*-MODY and only recently studies showed that patients with gene deletion have lesser renal impairment in comparison to patients with gene mutations (Dubois-Laforgue et al., 2017). The most consistent clinical feature in individuals with *HNF1B* mutations is the presence of renal abnormalities ranging from renal cysts, familial hypoplastic glomerulocystic kidney disease, renal malformations (i.e. single or horseshoe kidney) to atypical nephropathy (Edghill et al., 2006; Pearson et al., 2004). Insulin-dependent diabetes develops in 29% of *HNF1B* mutation carriers (Edghill et al., 2006), most frequently in the young age (median 20 years, range 15 days-61 years) (Edghill et al., 2006). Other symptoms are also possible and may comprise: neurological features, abnormal liver function, pancreatic hypoplasia, genital tract malformation, hypomagnesemia, hyperuricemia, early-onset gout or pectus excavatum (Clissold et al., 2015; Dubois-Laforgue et al., 2014), short stature, cataracts, splenomegaly, or even spina bifida occulta (Hogendorf et al., 2015).

Pharmacogenomics interventions have been developed for patients with *HNF4A*-MODY, *HNF1A*-MODY, and neonatal diabetes caused by activating mutations of K_ATP_ (potassium ATP-sensitive) channel-encoding genes (Hattersley et al., 2018). For *HNF1B-*MODY to do so, a better understanding of the disease pathogenesis is still needed. One of the great tools to have a broad insight in the disease associated changes in multi-organ disease such as *HNF1B-*MODY, are metabolomics studies.

Thus, the aim of our study was to define the serum metabolomic fingerprint of *HNF1B* mutations independent of coexisting diabetes and kidney disease and to understand its role in the disease pathogenesis.

## Material and methods

### Patient recruitment and sample collection

All patients gave their written consent for participation in the project and its protocol has been approved by the Institutional Bioethics Committee of the Medical University of Lodz (RNN/ll0/ll/KE 14th June, 2011). Patients with mutations of the *HNF1B* gene (HNF1Bmut) were recruited from the *Polish Registry for Pediatric and Adolescent Diabetes—nationwide genetic screening for* monogenic diabetes (Małachowska et al., 2018).

Overall, we obtained 53 serum samples from patients in the *HNF1B* group (N = 10), *HNF1A* group (N = 10), non-dialyzed group (N = 10), dialyzed group (N = 13) and control group (N = 12). Two samples from the non-dialyzed group were hemolyzed and thus were withdrawn from all further analyses. The clinical characteristics of the profiling group was provided in Table S1. Twenty-one patients were males (40%) and the proportion of men and women was similar between groups (p = 0.1047). Dialyzed patients were significantly older (Median 62.33 IQR [interquartile range] 54.85–66.18, all p values for comparisons with dialyzed patients < 0.0002), which is in-line with the natural course of chronic kidney disease, as end-stage renal disease usually develops after many years of kidney disease. There was no other significant age difference between *HNF1B*mut patients and other comparative groups (all p values > 0.15). Patients’ BMI (body mass index) did not differ significantly between the groups (p = 0.1127). The clinical characteristics of the group was provided in Table S1.

Having 53 samples and anticipated 630 metabolomic features (70% of 800–1000 features left after occurrence filtering) to test (alpha = 0.05/630 = 7.937E − 5), we expected to explain the variance equaling 0.8 (with error variance = 1) with 86% of power [calculations done by GPower software (Faul et al., 2009)].

The validation group for LPA and autotaxin measurements consisted of patients with *HNF1B*mut (N = 10), patients with the *HNF1B*-MODY phenotype referred for genetic testing but negative for *HNF1B*mut in whole-genome sequencing (N = 5), two groups of monogenic diabetes subtypes: *HNF1A*mut (N = 11) and *GCK*mut (N = 16), and healthy controls (N = 17). Patients were matched by age to the *HNF1B*mut group (except for *HNF1B*-negative group assembled from mostly newly diagnosed patients). The clinical characteristics of the validation group was provided in Table S2.

### Metabolomics studies

All metabolomics analyses were performed in compliance with current standards for MS-based metabolomics, with the use of quality control (QC) methodology (Daniluk et al., 2019). The identity of compounds that were found to be significant in class separation was confirmed by LC–MS/MS by using a QTOF (model 6550, Agilent) (as described before) (Daniluk et al., 2019). Details are described in the Supplementary Material—Molecular Methods. Identification level was provided in Table S3. Subsequent validation using ELISA was performed applying kit no. CEK623Ge (Cloude-Clone, Wuhan, China). The detection range was 123.5–10,000 ng/ml, sensitivity—52.7 ng/ml and intra-assay: CV < 10%, inter-assay: CV < 12%.

### Cell line experiments

It is suspected that the mutated variant other than that in the terminal exon leads to transcript degradation via the nonsense-mediated decay pathway, and the degradation rate depends on the type of mutation (Harries et al., 2005; Khajavi et al., 2006). Thus, the principal models for studying the *HNF1B*-MODY phenotype was created by using shRNA against Hnf1b and the effect is convergent with human missense or nonsense mutations. Therefore, we assumed that silencing of *HNF1B* expression would be a good surrogate versus mutating the gene.

In order to quantify the expression of *ENPP2 *in vitro HEK293 cells and HepG2 cells had their *HNF1B* gene silenced with the protocol described earlier (Fendler et al., 2016). Efficiency of *HNF1B* silencing by shRNA was shown in Fig. S1. RNA was isolated with RNeasy Mini Kit (Qiagen, Hilden, Germany) according to the manufacturer’s protocol with an additional step of DNase digestion with RNase-Free DNase Set (Qiagen, Hilden, Germany). Expressions of *ENPP2* target genes and *ACTB* were measured in duplicates with TaqMan™ Gene Expression Master Mix and TaqMan Assays: ENPP2-Hs00905125_m1; ACTB-Hs01060665_g1; (Applied Biosystems, Foster City, CA, USA) in accordance with the manufacturer’s instructions.

As the liver having high expression of *Hnf1b* is also one of the main tissues involved in the metabolism of LPA from serum*,* we decided to study the LPA stimulation of liver cells with the knockdown expression of *HNF1B.* Additionally liver dysfunction is a part *HNF1B-*MODY syndrome phenotype. To evaluate the impact of LPA treatment under different Hnf1b function, 100 nM of small interfering RNA (siRNA) targeting human *HNF1B* or negative control siRNA (Silencer Select #4392420 and #4390843, Ambion, Life Technologies) were transfected into HepG2 cells for 48 h with T-028 program by Nucleofector machine according to the manufacturer’s recommendations (Lonza, Basel, Switzerland). To analyze the effect of LPA, HepG2 cells were exposed to 10 µM albumin-bound sodium salt of 1-oleoyl lysophosphatidic acid (18:1-LPA) for the last 24 h prior to sample collection. A BSA supplemented medium was used for control conditions. Western blot was used to quantify the impact of LPA on autotaxin, β-actin and phosphorylated/dephosphorylated GSK3α/β proteins.

Finally, transcriptomic analysis was performed using Affymetrix Human Genome U133A 2.0 Array at GeneChip® Scanner 3000 7G. All reagents and equipment were obtained by Affymetrix/ThermoFisher Scientific (Waltham, MA, USA) and performed at the Department of Molecular Biology, Medical University of Silesia.

Details were presented in the Supplementary Material—Molecular Methods.

### Ingenuity pathway analysis

To evaluate the associated diseases and biological functions between the identified metabolite and *HNF1B*, we used QIAGEN’s Ingenuity® Pathway Analysis (IPA®, QIAGEN Redwood City, www.qiagen.com/ingenuity).

### Data analysis

#### Metabolomics

As the first step, the data was processed by the Molecular Feature Extractor (MFE) of the MassHunter Workstation software.

The abundance of molecular features was log-transformed before statistical analysis. The fold change of compound concentration was calculated as follows:

$$10^{{\left[ {\left( {\log _{{10}} {\text{mean}}\,{\text{metabolite}}\,{\text{concentration}}\,{\text{in}}\,{\text{Group}}\,1} \right) - \left( {\log _{{10}} {\text{mean}}\,{\text{metabolite}}\,{\text{concentration}}\,{\text{in}}\,{\text{Group}}\,2} \right)} \right]}}$$

For further analysis, we chose metabolic features detected in more than 80% of samples from each group. Then, we selected metabolic features with significantly different levels between groups, p value < 0.05 (ANOVA), after the adjustment for multiple comparison with the Benjamini–Hochberg correction and with the fold change > 3 or < 0.33 for comparison between *HNF1B*mut patients and non-dialyzed ones. Those metabolic features were then further identified. Profile analysis was performed by hierarchical clustering with Euclidean distance as a measurement of similarity of identified metabolites profiles. From identified metabolites, we selected those with the consistent fold change for *HNF1B* vs all four comparative groups (all FC > 2 or all < 0.5). A flowchart of *HNF1B*mut- specific metabolites selection was shown in Fig. S2. ROC curve analysis was then performed to find metabolites best discriminating *HNF1B*mut patients from other groups.

#### Transcriptomics

Statistical analysis of transcriptomic data was performed with GenePattern platform (Reich et al., 2006), MultiExperiment Viewer (MeV) software (Dana Farber, Boston, USA), STATISTICA 13.1 software (TIBCO Software, Palo Alto, CA) and Gene Set Enrichment Analysis (GSEA) (Subramanian et al., 2005) with molecular database c2cp (Liberzon et al., 2011). Gene-set permutation was used along with weighted enrichment statistics and Signal2Noise metric for ranking the genes. P values lower than 0.05 (after the adjustment for the Benjamini–Hochberg multiple comparison correction) were considered statistically significant.

## Results

### Metabolomic profiling of *HNF1B-*MODY syndrome

**Fig. 1:**
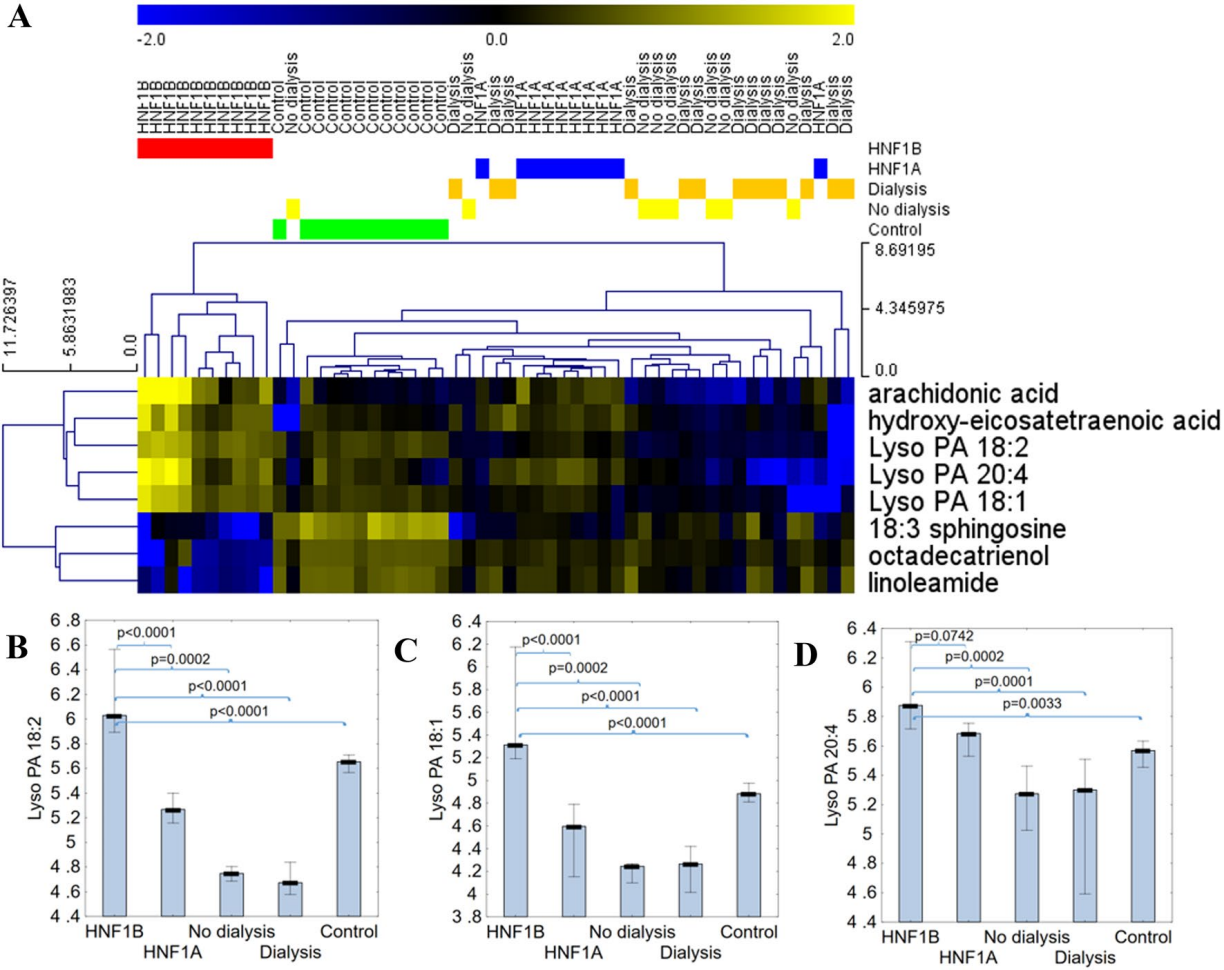
Identification of LPA as metabolic markers specific for *HNF1B*mut patients. **A** Hierarchical clustering with Euclidean distance and complete linkage as a measure of similarity of 8 identified metabolites and 53 participants’ serum samples. Rows were standardized. **B**–**D** Level of three lysophosphatidic acid derivatives [LysoPA (18:2) (**B**), LysoPA (18:1) (**C**), LysoPA (20:4) (**D**)] in patients with *HNF1B*mut and all four control groups (polycystic kidney disease, both non-dialyzed and dialyzed, *HNF1A*mut, healthy controls). Logarithmic scale was used. Medians and interquartile ranges were presented. Mann–Whitney U test with Bonferroni correction was used

Metabolomic fingerprint analysis yielded 952 metabolic features isolated from the positive ionization and 1004 from negative ionization mode. Out of those, 56 metabolic features met the criteria to enter the identification procedure (Fig. S2). Eventually, we identified 13 compounds from the positive and 14 from negative ionization mode (Table S3). Of 27 identified metabolites (Table 1), eight compounds met the fold change criteria (consistent FC for comparison with *HNF1B*mut group and > 2 or < 0.5 vs all other comparative groups). We showed that the obtained panel of 8 metabolites perfectly separated patients with *HNF1B*mut from all other comparative groups (Fig. 1A). AUC above 0.9 was achieved by six metabolites—arachidonic acid (higher levels among *HNF1B*mut patients), hydroxyeicosatetraenoic acid (higher level), and linoleamide (lower level) and three lysophosphatidic acids [LPA (18:2), LPA (18:1) and LPA (20:4)] (Fig. 1B–D) which proved to best separate samples of patients with *HNF1B*mut (Table S4). Thus, in further studies, we focused on linking the LPA with *HNF1B-*MODY syndrome pathogenesis, searching for a source of higher LPA level in serum among *HNF1B*mut patients and on studying the effect of higher LPA level on *HNF1B* deficient cells.

**Table 1:** All identified metabolites from metabolic features that had ANOVA p values lower than 0.05 (after the adjustment for the Benjamini–Hochberg multiple comparison correction)

Compound name	FC vs HNF1a	FC vs non-dialyzed	FC vs dialyzed	FC vs control	Adjusted p
Lyso PC (15:0)	1.37	4.30	4.56	2.39	7.774E − 11
Lyso PAF C-18	1.37	6.33	7.56	3.53	1.446E − 10
Lyso PE (18:0)	1.01	4.51	4.60	2.30	3.341E − 10
Lyso PC (16:1)	1.68	6.04	5.78	2.98	7.893E − 10
Lyso PC (16:0)	1.42	6.13	6.05	3.13	7.932E − 10
Lyso PI (18:0)	1.01	5.12	5.57	2.19	1.519E − 09
Arachidonic acid	2.24	6.74	5.93	3.79	1.557E − 09
PC (22:1)	1.66	5.78	6.66	3.31	8.838E − 09
Sphingosine (18:3)	0.39	0.29	0.34	0.11	1.534E − 06
Linoleamide	0.03	0.05	0.03	0.01	1.542E − 05
p-cresol sulfate	0.62	0.26	0.08	0.46	1.582E − 05
Lyso PA (20:4)	2.64	5.68	8.29	2.93	2.390E − 05
Octadecatrienol [fatty alcohol (18:3)]	0.01	0.02	0.02	0.01	5.882E − 05
Palmitoleamide	0.06	0.28	0.30	3.02	8.028E − 05
p-cresol	0.75	0.27	0.08	0.46	8.832E − 05
Lyso PA (18:2)	8.86	26.95	135.55	3.48	2.093E − 04
Androsterone sulfate	1.55	2.04	7.21	2.29	4.649E − 04
Bilirubin	14.82	0.21	0.47	0.30	7.947E − 04
PC (21:1)	1.73	4.78	20.56	3.54	1.311E − 03
Lyso PE (17:2)	0.80	19.34	4.73	1.91	1.888E − 03
Lyso PC (17:0)	1.31	3.81	1.98	1.54	7.998E − 03
Lyso PA (18:1)	34.53	83.55	93.23	5.38	9.059E − 03
Lyso PE (16:0)	0.88	4.74	25.96	2.08	1.242E − 02
Lyso PI (16:0)	1.17	3.66	10.95	1.91	1.316E − 02
Fatty alcohol (18:4)	0.45	5.05	0.61	0.22	1.420E − 02
Lyso PC (18:2)	1.02	4.33	9.40	1.53	1.745E − 02
Hydroxy-eicosatetraenoic acid	4.57	102.63	64.16	21.79	2.783E − 02

FCs (Fold changes) for comparison of *HNF1B* groups with all other groups were given

### In silico links between LPA and RCAD syndrome pathogenesis

**Fig. 2:**
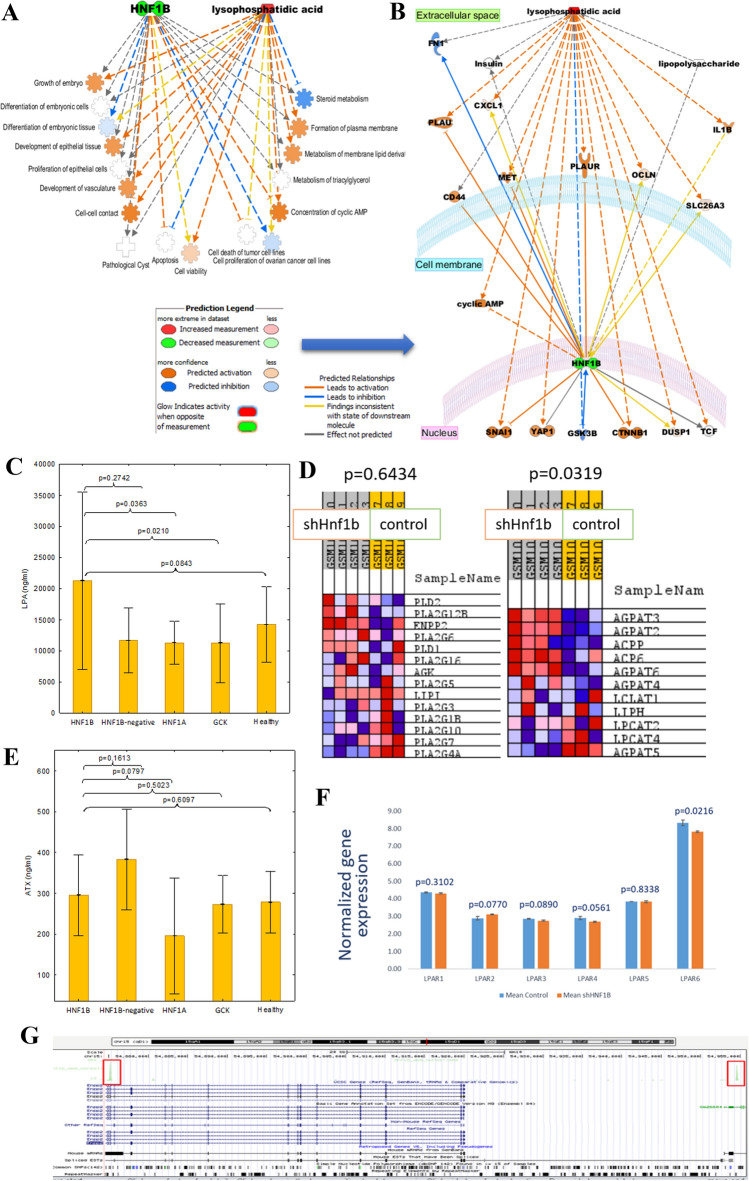
Molecular associations between *HNF1B* and LPA molecules. **A**, **B** Functional analysis of *HNF1B* gene and lysophosphatidic acid run by Ingenuity Pathway Analysis. Diseases and functions (**A**) associated with both *HNF1B* gene and lysophosphatidic acid were presented. Connection of both compounds with pathological cyst formation, proliferation of epithelial cells and development of epithelial tissues suggests that lysophosphatidic acid may be involved in cyst formation in *HNF1B*-deficient organisms. **B** Molecular network analysis of *HNF1B* transcription factor and lysophosphatidic acid. Molecular function prediction tool was used to predict the effect of upregulated LPA and downregulated *HNF1B* on molecules in the network. **C** ELISA measurements of serum LPA in validation group. *T*-test was used. **D** Gene set enrichment analysis of genes associated with LPA production (left) and degradation (right) analyzed with data from Kornfeld et al., 2013, GSE42188. **E** ELISA measurements of serum autotaxin in validation group. *T*-test was used. **F** Altered expression of LPA receptors in the mice model of *HNF1B*-MODY (Kornfeld et al., 2013, GSE42188). **G** Hnf1b binding in *ENPP2* gene region—immunoprecipitation from Aboudehen et al., 2016, GSE71250

To search for other molecular interactions between *HNF1B* and LPA, we used in silico Ingenuity Pathways Analysis software. LPA and *HNF1B* are involved in many common diseases and functions (Fig. 2A) including developmental processes (in particular—epithelial ones), lipid metabolism as well as cell proliferation and death. This is in-line with the theory that cysts in RCAD have an epithelial origin. What is more, many processes associated with lipid metabolism i.e. metabolism of membrane lipid derivatives, steroid metabolism, or metabolism of triacylglycerol, were associated with both LPA and *HNF1B*. The most interesting was the association of both elements with pathological cyst formation. Several interesting molecules including insulin, *TCF* and three genes associated with Wnt/beta-catenin signaling, *CTNNB1*, *CD44* and *GSK3B,* were found associated with both LPA and *HNF1B* (Fig. 2B). Wnt/beta-catenin signaling pathway is suspected to be responsible for cyst growth in *HNF1B-*MODY. This net of interaction with Wnt/beta-catenin signaling pathway provides a basis for a possible mechanism of LPA role in syndrome pathogenesis.

### Altered LPA synthesis and degradation pathways in *HNF1B* deficient models

The main pathways of LPA synthesis in serum occur via a circulating enzyme autotaxin (ATX) encoded by the *ENPP2* gene (Nakanaga et al., 2010). To validate our metabolomic profiling results, we collected new serum samples from the following groups of patients: *HNF1B*-mutated patients, *HNF1A-*mutated patients, *GCK-*mutated patients (as the third type of MODY phenotypes), *HNF1B-*negative patients (patients referred for *HNF1B* genetic testing due to *HNF1B*-MODY phenotype but with negative results confirmed by Whole-Genome-Sequencing) and control healthy individuals. We confirmed that patients with the mutations within the *HNF1B* gene had a higher level of LPA than *HNF1A*-mutated, *GCK*-mutated patients (Fig. 2C) and healthy individuals (at borderline significance). The autotaxin (Fig. 2E) level, however, was not significantly elevated; thus, we assumed that factors other than a higher ATX level in serum are responsible for a higher level of LPA in the sera of *HNF1B*-MODY patients. Therefore, we measured the expression of the *ENNP2* gene in two cell lines: hepatic (HepG2) and kidney (HEK293) with a knockdown expression of *HNF1B* (Fig. S3)*.* Somewhat surprisingly, we observed downregulation of the *ENPP2* gene with borderline significance, but no up-regulation. To explain what could cause this drop of expression we reanalyzed chromatin immunoprecipitation data of *HNF1B* (Aboudehen et al., 2016) and found two binding peaks near the *ENPP2* gene which showed that expression of the *ENPP2* gene can be regulated by transcriptional factor *HNF1B* explaining the downregulation of *ENPP2* in our experiment due to the loss of *HNF1B* (Fig. 2G). Subsequently, we searched for genes, other than *ENPP2,* which are associated with LPA production and degradation. This led us to create two new gene sets which we then tested on transcriptomic data of livers from mice with knockdown expression of *HNF1B* (Kornfeld et al., 2013)*.* Genes associated with LPA synthesis were not upregulated significantly (Fig. 2D). However, the LPA degradation gene set was significantly upregulated. This suggests that within an organism with globally downregulated expression of *HNF1B,* the liver—the main organ responsible for LPA uptake and degradation—overexpresses genes able to degrade LPA, thus counteracting its higher serum level. Moreover, the expression of LPA receptors was also affected by *HNF1B* knockdown (Fig. 2F) with the highest expressed receptor in liver—*LPAR6*—being downregulated due to Hnf1b loss and thus protects cells from LPA overstimulation.

### LPA stimulation effect on cells with *HNF1B* knockdown

**Fig. 3:**
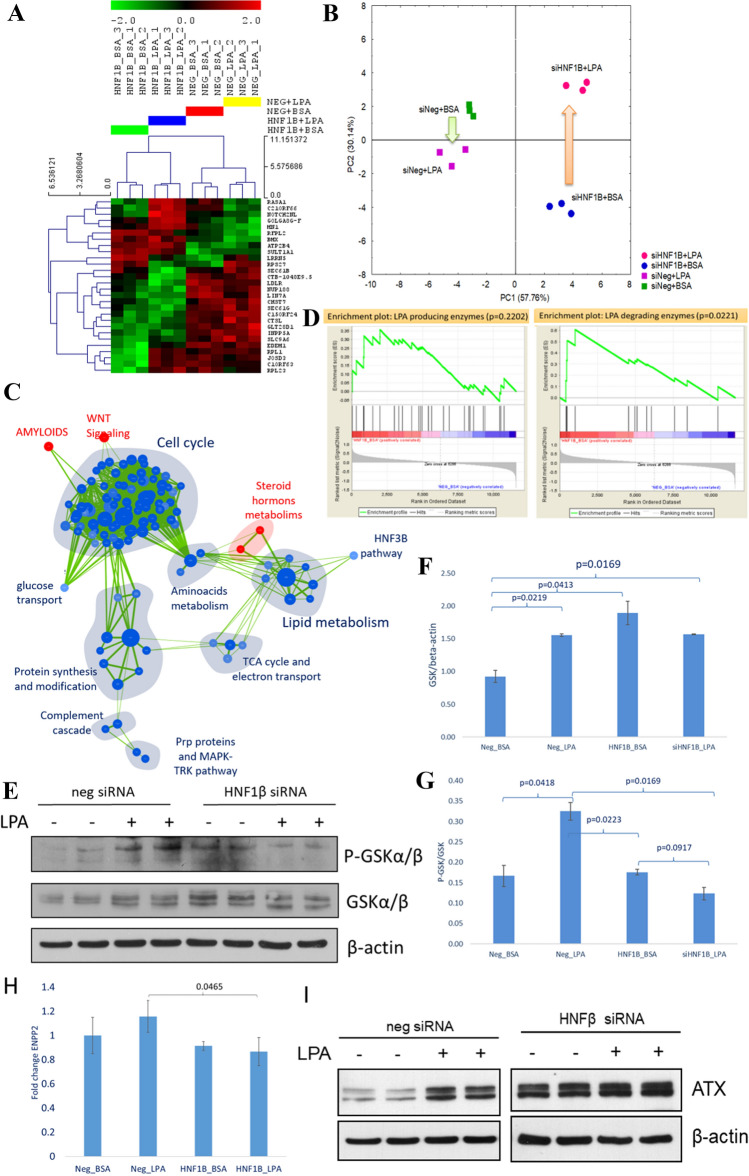
Molecular effect of LPA stimulation on hepatic cells with *HNF1B* knockdown. **A**, **B** Hierarchical clustering (**A**) and principal component analysis (**B**) of genes significantly affected by LPA stimulation and *HNF1B* knockdown (FDR for ANOVA < 0.15) (n = 3 per group). **C** Enrichment map visualization of gene sets significantly upregulated (red) and downregulated (blue) with LPA stimulation and *HNF1B* knockdown [vs unstimulated cells with a normal level of *HFN1B* expression (Control)]. **D** Gene set enrichment analysis of genes associated with LPA production and degradation in cells with *HNF1B* knockdown (vs control). **E**–**G** GSKalpha/beta protein and its phosphorylated form level increase with LPA stimulation and knockdown of *HNF1B.* Cells with *HNF1B* knockdown do not respond to LPA stimulation (n = 2 per group). **H**, **I** Autotaxin RNA level (*ENPP2* gene, H) is not elevated with *HNF1B* knockdown (n = 3) but its protein level is increased with both *HNF1B* knockdown and LPA stimulation (n = 2) (**I**)

To measure the effect of LPA stimulation on *HNF1B* deficient cells, we used cell culture of HepG2 cells with *HNF1B* knockdown*,* which was then stimulated with LPA. Transcriptomic analysis (Table S5) revealed 28 significantly affected genes (Fig. 3A). Principal component analysis (PCA) of those genes (Fig. 3B) showed that the first component is responsible for the difference caused by *HNF1B* knockdown (PC1 = 58%), and the second component reflects mainly differences caused by LPA stimulation (PC2 = 30%). Interestingly, the effect of LPA stimulation on normal hepatic gene expression seemed to exert an opposite effect on that observed in cells with *HNF1B* knockdown*.* In order to study in details the effect of LPA stimulation combined with *HNF1B* knockdown, we performed a gene set enrichment analysis with enrichment map as a visualization (Fig. 3C). Most of the significantly altered pathways were downregulated (blue), including those associated with glucose transport, lipid metabolism and cell cycle. Only three pathways were upregulated: Steroid hormones metabolism, Amyloids, and Wnt signaling. We checked our LPA producing and degrading gene sets (Fig. 3D) and we confirmed that the LPA production pathway was not significantly altered but the one for LPA degradation was significantly upregulated. In order to validate our in silico predictions, we performed a Western blot analysis of GSK-3alpha/beta level and its phosphorylated form (Fig. 3E–G). In normal cells, LPA stimulation increased both the level of GSK-3alpha/beta and the ratio of its phosphorylated form. However, in cells with *HNF1B* knockdown, we did not observe an increase in GSK-3alpha/beta, and its phosphorylated form was even marginally downregulated (p = 0.0917). This indicates that cells with *HNF1B* knockdown may be less responsive to LPA stimulation and higher level of LPA in the serum might be a compensatory mechanism for some tissues requiring LPA also having detrimental effect on tissues sensitive to LPA with different LPA receptor profile (not regulated by Hnf1b).

Additionally, once again, we checked the mRNA level of the *ENPP2* gene (Fig. 3H); it was downregulated (non-significantly) by *HNF1B* knockdown. In control cells, the LPA stimulation increased the expression level of *ENPP2* but in cells with *HNF1B* knockdown this effect was not observed and *ENPP2* levels were significantly lower than in normal cells stimulated with LPA. The effect of LPA stimulation on autotaxin protein level in normal cells was confirmed with Western blot analysis (Fig. 3I). However, the protein level of autotaxin in hepatic cells with *HNF1B* knockdown (without LPA) was much greater than in normal cells and this finding can explain the elevated level of LPA observed in *HNF1B*mut patients. This suggests the post-transcriptional regulation of autotaxin level in cells with deficient *HNF1B*.

Altogether, this series of experiments showed that the effect of LPA stimulation under normal or down-regulated levels of *HNF1B* produced a different biological effect. Patients with *HNF1B*mut have higher levels of LPA despite unaltered levels of serum autotaxin, the presence of *HNF1B*-deficiency is associated with lower autotaxin RNA in liver, but higher at the protein level. The overabundance of LPA is likely mitigated by a downregulation of the main hepatic LPA receptor (LPAR6) (or is a culprit of it). Also, we observed the upregulation of LPA-degrading pathways in liver which could help remove the excess of LPA, however not efficiently. The resulting overabundance of LPA constantly overstimulates the cells despite countermeasures, most likely through disturbing the Wnt/GSK-3 signaling [likely due to competitive binding to chromatin site between Hnf1b and beta-catenin/lymphoid enhancer binding factor (Chan et al., 2019)], which may thus contribute to the development of *HNF1B-*MODY phenotype.

## Discussion

Our study found that the LPA level was elevated in the serum of patients with *HNF1B*mut likely due to the increased protein levels of the main LPA-producing enzyme (autotaxin) in liver cells*.* LPA may contribute to the development of *HNF1B-*MODY phenotype by affecting Wnt/GSK-3 signaling.

As far as we are concerned, this is the first study reporting changes in serum metabolites of *HNF1B*mut patients but metabolomics studies in other MODY types were previously performed. An analysis by Gloyn et al. aimed to determine the metabolic profile of patients with monogenic diabetes (2012), but patients with *HNF1B* mutations were not included in that study. Even though the patients had distinct biochemical pathways affected, it was not possible to find any urinary biomarkers which could distinguish patients with *GCK*-MODY and *HNF1A*-MODY from patients with type 2 diabetes. Recent study by Juszczak et al. found an altered cortisol metabolism in *HNF1A*-MODY patients (vs type 2 diabetes patients and healthy controls) by means of targeted gas chromatography–mass spectrometry analysis (2020). Another metabolomic study in the monogenic diabetes field was aimed at *GCK* gene mutation carriers (Spégel et al., 2013). The analysis revealed that *GCK-*MODY patients have a more similar metabolomic pattern to healthy individuals than to patients with other forms of diabetes (*HNF4A-*MODY, *HNF1A-*MODY and type 2 diabetes). In our study, we found eight metabolites that serum changes were specific to *HNF1B*mut. However, due to the low prevalence of *HNF1B*mut and continuous drop in the price of gene sequencing and high price of HPLC–MS measurements with internal standard, the biomarker approach for detecting rare molecular aberrations seems outdated. Nevertheless, metabolomics profiling provides deep insight into disease biology, may help understand molecular causes of symptom development and may eventually help select new drug targets for rare diseases.

The only study using metobolomics in *HNF1B*-MODY research was performed in mice. Torell et al. used the metabolomics approach to study differences in metabolites in the mice model of *HNF1B*-MODY vs wild type in different tissues (2018). Due to the difference in used techniques (GC–QTOF–MS vs HPLC–QTOF–MS), the overlap between their and our study is small, but they found arachidonic acid to be elevated in livers and kidneys of *HNF1B-*MODY mice which could suggest that those two tissues may be the source of serum elevated arachidonic acid found in our study. Additionally, arachidonic acid was previously found to affect the genital tract development, thus its involvement in the *HNF1B-*MODY phenotype pathogenesis would be interesting for further studies (Goldman, 1987).

Our main findings considered the elevated level of LPA and its possible involvement in syndrome pathogenesis. LPA is a highly bioactive phospholipid and its serum concentration is thought to depend on the activity of an enzyme called autotaxin (Tanaka et al., 2006). Elevated levels of autotaxin were previously associated with liver injury, chronic hepatitis and liver cirrhosis (Watanabe et al., 2007a, 2007b). Serum autotaxin was also found to be an indicator of the severity of liver disease and the prognosis of cirrhotic patients (Pleli et al., 2014). As the altered hepatic function is one of the symptoms of *HNF1B-*MODY, so it may be one of the possible mechanisms underlying the phenomenon of elevated LPA level among those patients, however in our case not in serum but directly within liver cells.

LPA is interesting not as a biomarker of *HNF1B*-MODY but mostly because of its biological functions. It is known that serum levels of LPA are higher than plasma levels, and platelet activation leads to an increased LPA production (Sano et al., 2002). However, there are differences in levels of molecular forms of LPA found in different sources. In resting and thrombin-stimulated platelets, LPA forms ranks in the following order (from most abundant) 16:0 > 18:0 > 20:4 > 18:1 > 18:2; in plasma: 18:2 > 18:1 > 18:0 > 16:0 > 20:4; whereas in serum: 20:4 > 18:2 > 16:0 > 18:1 > 18:0. In our study, top changed LPAs were 18:1 and 18:2, which strongly suggests that the differences are coming from plasma, not platelets, and as such are constantly present in patients’ bloodstream and may modify the HNF1B-MODY phenotype.

Interestingly, in our analysis, patients with *HNF1B* deletion seem to have a slightly higher level of all LPA forms [LPA (18:1) p = 0.1807, LPA (18:2) p = 0.1191, LPA (20:4) p = 0.0863]. However, as this comparison is underpowered (4 vs 6 patients), the confirmation on a bigger group or in vivo studies would be required.

LPA was found to be a lipid modulator of cyst growth in adult dominant polycystic kidney disease (Blazer-Yost et al., 2011). Blazer-Yost et al. identified LPA as a component of cyst fluid and serum that stimulates secretory Cl^−^ transport in the epithelial cell type which lines renal cysts. As LPA was shown to be responsible for cyst growth, the scientists patented the usage of LPA inhibitor for treating cystic diseases. Using the *Ingenuity Pathway Analysis* we found that LPA is involved in many pathological processes affected by mutations in *HNF1B*, i.e. proliferation of epithelial cell, cell viability and apoptosis (Fig. 2F).

Some theories of cyst formation involve planar cell polarity phenomenon. Planar cell polarity and its manifestation, oriented cell division (OCD), are important mechanisms responsible for proper tissue growth. OCD was found to control, at least in part, kidney tubule elongation (Fischer et al., 2006). Altered OCD was found to be a prelude to cyst formation (Fischer et al., 2006). Lokmane et al. found that Hnf1b directly regulates the expression of Wnt9b and thus alters planar cell polarity which leads to cyst formation (Lokmane et al., 2010). There is also evidence that LPA is able to activate Wnt/β-catenin signaling through activation of β1-integrin (Burkhalter et al., 2015). However, it remains unknown whether the interplay between high LPA, low *HNF1B* and Wnt/β-catenin pathway is a culprit of cyst formation in *HNF1B*-MODY syndrome. We showed that GSK-3alpha-beta phosphorylation in response to LPA stimulation is disturbed in cells with *HNF1B* knockdown.

Due to the widespread of LPA receptors in various cells, its elevated level may contribute also to other symptoms of *HNF1B-*MODY syndrome by affecting cells in the nervous system (neural progenitor cells, astrocytes and oligodendrocytes), reproductive system and proliferating pre-adipocytes (Sheng et al., 2015).

Despite the fact, that in our study we focused mainly on LPA, other metabolites found in our metabolomics profiling may be important for *HNF1B-*MODY pathogenesis, such as linoleamide, an endogenous sleep inducing lipid, greatly downregulated in *HNF1B-*MODY, which was found to release Ca^2+^ in renal tubal cells (Huang & Jan, 2001) from the endoplasmic reticulum. Hydroxyeicosatetraenoic acid—found to be elevated among *HNF1B-*MODY patients was previously linked with diabetes-induced endothelial dysfunction (Otto et al., 2020).

The main limitation of our work is the relatively small sample size and lack of external cohort validation. However, as we validated our results with ELISA technique in independent samples and the disease itself being very rare, we believe this problem is partially mitigated.

Further studies are required, especially in vivo*,* to explain the role of LPA in *HNF1B-*MODY pathogenesis and the crucial question to be answered is whether the inhibition of LPA can affect cyst growth in the patients with mutated *HNF1B.*

## Conclusions

Patients with the mutations in the *HNF1B* gene are characterized by a higher level of LPA in serum. The elevated level of LPA may contribute to the development of the *HNF1B*-MODY phenotype.

## Supplementary Information

Below is the link to the electronic supplementary material.

Supplementary file1 (XLSX 2114 KB)

Supplementary file2 (DOCX 24 KB)

## Data Availability

All raw data are attached to the manuscript in Supplementary materials and via the repository: https://biostat.umed.pl/public_ftp/bmalachowska/
