# Supplementary material for: Elevated level of lysophosphatidic acid among patients with HNF1B mutations and its role in RCAD syndrome: a multiomic study
Source: Metabolomics. 2022 Feb 18;18(3):15. doi: 10.1007/s11306-022-01873-z (PMC8857088; doi:10.1007/s11306-022-01873-z)
Supplement: Supplementary file 2 — Supplementary file2 (DOCX 24 KB) [file 11306_2022_1873_MOESM2_ESM.docx]

## Serum sample preparation

During each blood withdrawal, we collected one blood sample of 4.0 mL into a tube with silica as a clot activator (Becton Dickinson, New Jersey, NJ, USA)[18]. Serum was separated from the erythrocyte mass by centrifugation (10 min, 800 x g) and then frozen in 0.5mL Eppendorf tubes in –80^o^C until metabolite concentration measurements were performed.

Protein precipitation and metabolite extraction were performed by the addition of 1 volume of serum to 3 volumes of cold (–20ºC) mixture of methanol and ethanol (1:1). Samples were then vortex-mixed and stored at 4ºC for 10 min. The pellet was removed by centrifuging at 21 000 × g for 20 min at 4ºC, and the supernatant after filtration through a 0.22 µm nylon filter was ready for analysis.

Quality control (QC) samples were prepared by pooling equal volumes of serum from each sample. QC was injected at the beginning of the run and after every five - six real samples to provide a measurement not only of the system’s stability and performance but also of the reproducibility of the sample treatment procedure. The obtained mixture was prepared following the same procedure as the remainder of the samples.

## Metabolomic analysis and metabolite identification

Samples were analyzed by an LC-MS system consisting of 1290 Infinity UHPLC with a degasser, two binary pumps and thermostated autosampler coupled to a 6550 Q-TOF-MS detector (both Agilent Technologies, Santa Clara, CA, USA). 1µL of extracted serum sample was injected to a reversed-phase column (Zorbax Extend-C18, RRHT, 50 ×2.1 mm, 1,8µm; Agilent Technologies) thermostated at 60oC. The flow rate was 0.6 mL/min with solvent A - water with 0.1% formic acid, and solvent B - acetonitrile with 0.1% formic acid. The gradient started from 5% B for the first minute. Next, the mobile phase composition was changed by increasing B to 80% (from 1 to 7 minutes) and to 100% (from 7 to 11.5 minutes). and returned to starting conditions in 0.5 min, keeping the re-equilibration at 5% B for 3 min. Data were collected in positive and negative ion modes operated in full scan mode from 50 to 1000 *m/z* with a scan rate of 1,5 scan per second. Accurate mass measurements were obtained by means of an calibrant solution delivery using a dual-nebuliser ESI source that continuously introduces a calibrating solution which contains reference masses at *m/z* 121.0509 (protonated purine) and *m/z* 922.0098 (protonated hexakis(1H,1H,3H‐tetrafluoropropoxy)phosphazine or HP‐921 ) for positive and *m/z* 119.0363 (proton abstracted purine) and *m/z* 966.0007 (formate adduct of HP-921) for negative ionisation mode. The capillary voltage was set to -3000 V for positive and 4000 V for negative ionisation mode; the drying gas flow rate was 12 L/min at 250°C and gas nebulizer at 52 psig;. Samples were analyzed in a randomised order in two runs (first for positive and second for negative ion mode). At the beginning of each run, a batch of fifteen injections of a QC sample was used to condition the column.

Experiments were repeated with identical chromatographic conditions like in the primary analysis. Ions were targeted for collision-induced dissociation (CID) based on the previously determined accurate mass and retention time. A comparison of the structure of the proposed compound with the fragments obtained can confirm the identity. Accurate mass data and isotopic distributions for the precursor and product ions were studied and compared to spectral data of reference compounds, if available, obtained under identical conditions for final confirmation (HMDB, METLIN, Lipidmaps, KEGG).

As the first step of data analysis, the metabolomics data was processed by the Molecular Feature Extractor (MFE) of the MassHunter Workstation software.

The abundance of molecular features was log-transformed before statistical analysis. The fold change of compound concentration was calculated as follows:

$${10}^{(\left( {log}_{10}mean metabolite concentration in Group 1 \right)-\left( {log}_{10}mean metabolite concentration in Group 2 \right))}$$

## RNA extraction and qPCR protocol

The concentration and quality of isolated RNA samles were determined by ultraviolet spectrophotometry (NanoDrop 8000; Thermo Fisher Scientific, Waltham, MA, USA). A total of 1µg of RNA was used for cDNA synthesis using the High-Capacity cDNA Reverse Transcription Kit (Thermo Fisher Scientific, Waltham, MA, USA) and 80 ng of cDNA was used for each qPCR reaction. The expression of ENPP2 target gene and ACTB as a reference genes were measured in duplicates with TaqMan™ Gene Expression Master Mix and TaqMan Assays: ENPP2 - Hs00905125_m1; ACTB - Hs01060665_g1; (Applied Biosystems, Foster City, CA, USA) in accordance with the manufacturer’s instructions. The reaction was performed using Mx3000P qPCR System (Agilent Technologies, Santa Clara, CA, USA). For the analysis of qPCR data, the relative gene expressions (FC – fold change) were calculated using cycle threshold (Ct) values and following formulas:

$${\Delta Ct=Ct}_{ENPP2}-{meanCt}_{ACTB}$$

$$FC=2^{-\Delta\Delta Ct}=2^{-(}{\Delta Ct}_{siRNA}-{\Delta Ct}_{Neg})$$

## Cell culture protocol

The human hepatoma cell line (HepG2; ECACC, Salisbury, UK) was cultured in high-glucose Dulbecco’s modified Eagle’s medium (DMEM, Biowest) supplemented with 10% (v/v) heat inactivated fetal bovine serum (FBS; Gibco, Life Technologies), 2 mM L-glutamine (Gibco, Life Technologies), 1 mM sodium pyruvate (Gibco, Life Technologies) and antibiotics (100 U/mL penicillin and 100 µg/mL of streptomycin, Gibco, Life Technologies). The cultures were maintained at 37°C in a 95% humidified 5% CO_2_ atmosphere.

## Hnf1b knockdown and LPA stimulation experiment including Western blot and transcriptomic analyses

For knockdown experiments, 100 nM of small interfering RNA (siRNA) targeting human HNF1β or negative control siRNA (Silencer Select #4392420 and #4390843, Ambion, Life Technologies) were transfected into HepG2 cells for 48 h with T-028 programme by Nucleofector machine according to the manufacturer’s recommendations (Lonza, Basel, Switzerland). To analyse the effect of LPA, HepG2 cells were exposed to 10 µM albumin-bound sodium salt of 1-oleoyl lysophosphatidic acid (18:1-LPA) for the last 24 hours prior to sample collection. A BSA supplemented medium was used for control conditions.

The human hepatoma cell line (HepG2; ECACC, Salisbury, UK) was cultured in high-glucose Dulbecco’s modified Eagle’s medium (DMEM, Biowest) supplemented with 10% (v/v) heat inactivated fetal bovine serum (FBS; Gibco, Life Technologies), 2 mM L-glutamine (Gibco, Life Technologies), 1 mM sodium pyruvate (Gibco, Life Technologies) and antibiotics (100 U/mL penicillin and 100 µg/mL of streptomycin, Gibco, Life Technologies). The cultures were maintained at 37°C in a 95% humidified 5% CO_2_ atmosphere.

After incubation, HepG2 cells were lysed in ice-cold lysis buffer that contained 50 mM Tris- HCl (pH 7.4), 1% Nonidet P-40, 1% Triton-X-100, 150 mM NaCl, 5 mM ethylenediaminetetraacetic acid (EDTA), 10 mM NaF, 1 mM phenylmethylsulfonyl fluoride, 1 mM NaWO 4 , 5 μg/ml pepstatin A, 10 μg/ml leupeptin, and 2 μg/μl aprotinine. The proteinconcentrations of the lysates were quantified by the Bio-Rad protein assay (Bio-Rad, Hercules, CA) with BSA as the standard. A total of 20 μg of protein lysates was loaded onto 10% SDS-PAGE gels, transferred to polyvinylidene difluoride (PVDF) membranes (Millipore) and subjected to immunoblot analysis using antibodies against autotaxin, GSKα/β, phosphorylated- GSKα/β (Ser21/9) and β-actin proteins. Bands were visualized using an enhanced chemiluminescence reagent (Thermo Scientific).

RNA isolation and RINs Total RNA was isolated from 5x10 5 cells resuspended in Trizol (Invitrogen, Carlsbad, California, USA) in accordance with Invitrogen protocol. Samples were treated with RNase-Free DNase (RNase-Free DNase Set, Qiagen, Hilden, Germany). RNA integrity was assessed using TapeStation 4200 (Agilent Technologies, Santa Clara, California, USA) based on RIN (RNA Integrity Number) value. Purity and concentration of RNA samples were measured using NanoDrop 8000 Spectrophotometer (Thermo Fisher Scientific, Waltham, Massachusetts, USA). Synthesis of cRNA was performed with GeneChip® 3' IVT PLUS Reagent Kit and washing and staining was performed with GeneChip® Hybridization, Wash, and
Stain Kit on GeneChip® Fluidics Station 450. Gene expression was measured with Affymetrix Human Genome U133A 2.0 Array at GeneChip® Scanner 3000 7G. All reagents and equipment were obtained by Affymetrix /ThermoFisher Scientific, Waltham, MA, USA) and performed at Department of Molecular Biology, Medical University of Silesia.
